# UCPSO: A Uniform Initialized Particle Swarm Optimization Algorithm with Cosine Inertia Weight

**DOI:** 10.1155/2021/8819333

**Published:** 2021-03-18

**Authors:** Jian Zhang, Jianan Sheng, Jiawei Lu, Ling Shen

**Affiliations:** ^1^School of Mechanical Engineering, Tongji University, Shanghai 200092, China; ^2^Shanghai University of Medicine & Health Sciences, Shanghai 201318, China

## Abstract

The particle swarm optimization algorithm (PSO) is a meta-heuristic algorithm with swarm intelligence. It has the advantages of easy implementation, high convergence accuracy, and fast convergence speed. However, PSO suffers from falling into a local optimum or premature convergence, and a better performance of PSO is desired. Some methods adopt improvements in PSO parameters, particle initialization, or topological structure to enhance the global search ability and performance of PSO. These methods contribute to solving the problems above. Inspired by them, this paper proposes a variant of PSO with competitive performance called UCPSO. UCPSO combines three effective improvements: a cosine inertia weight, uniform initialization, and a rank-based strategy. The cosine inertia weight is an inertia weight in the form of a variable-period cosine function. It adopts a multistage strategy to balance exploration and exploitation. Uniform initialization can prevent the aggregation of initial particles. It distributes initial particles uniformly to avoid being trapped in a local optimum. A rank-based strategy is employed to adjust an individual particle's inertia weight. It enhances the swarm's capabilities of exploration and exploitation at the same time. Comparative experiments are conducted to validate the effectiveness of the three improvements. Experiments show that the UCPSO improvements can effectively improve global search ability and performance.

## 1. Introduction

Since the particle swarm optimization algorithm (PSO) was proposed by Kennedy and Eberhart in 1995 [1], it has obtained great achievements in finding the optimal value of continuous nonlinear equations [2]. PSO is a special branch of evolutionary algorithms. It adopts social learning among swarms and the self-cognition of individuals to replace the common theory of evolution algorithms (EAs)-survival of the fittest [3]. The bionics mechanism of PSO gives PSO swarm intelligence [4] and enables PSO to imitate the complex search behaviour of swarms, such as bird swarms, fish schools, and ant colonies. Different from EAs, PSO has a simpler iteration mechanism and fewer control parameters [5]. Therefore, it is widely used in practical engineering areas. For example, PSO is usually applied to image processing [6], parameter optimization [7, 8], scheduling optimization [9], clustering [10], and price forecasting [11].

One of the main advantages of PSO is its easy implementation [12]. A large number of numerical experiments also prove that PSO has high convergence accuracy and a fast convergence speed [13]. Consequently, researchers have carried out numerous studies on PSO. However, some limitations of PSO have been found during long-term research work. When facing complex functions, PSO is troubled by falling into a local optimum or premature convergence [14]. Meanwhile, the performance of the original PSO is inadequate and must be improved. To solve these problems, many researchers have proposed improvements. These improvements mainly focus on PSO parameters, particle initialization, and population topology.

Inertia weight is a very important parameter in the PSO algorithm. It controls the balance between the two critical behaviours of PSO: global and local search. Researchers have created various forms of inertia weight. These forms of inertia weight enhance the performance of PSO. Shi and Eberhart proposed a parameter called inertia weight *ω* in PSO to balance exploration and exploitation [15]. The appearance of *ω* created a new way to improve the performance of PSO. Then, Eberhart and Shi introduced a linear decreasing inertia weight [16]. This linear decrease in inertia weight greatly improves the comprehensive performance of PSO. It relieves the problem of falling into a local optimum. Recently, Tian et al. adopted a multistage strategy to refine the change process of inertia weight. They split the curve of inertia weight into two stages to satisfy specific requirements. The variant proposed by them achieves excellent performance in the experiment [17].

The quality of the initial particles is related to the PSO results. Many works have been performed to distribute initial particles more dispersedly and make initial particles closer to the global optimum. Tian [18], Zhang [19], and Xu [20] adopted chaotic sequences for particle initialization to increase the diversity of initial particles. Chaotic initialization achieves certain success compared to random initialization under the same conditions. Rahnamayan [21] employed the symmetry strategy in swarm initialization. Symmetry initialization can prevent initial particles from being distant from the global optimum. DMPSO [22] combines chaotic initialization with opposition-based initialization. Experiments validate that the hybrid initialization can recognize the search area better. In addition, MCJPSO [23] randomly divides the entire search space and distributed particles over a search space in independent slots. This semirandom initialization can overcome the limitation of the original PSO. Rauf et al. used the Weibull probability sequence to generate numbers at random locations for swarm initialization. This method is able to enhance the diversity of swarms [24].

To enhance the global search ability and the comprehensive performance of PSO, a uniform initialized particle swarm optimization algorithm with cosine inertia weight (UCPSO) is proposed in this paper. UCPSO combines three effective improvements: an inertia weight in the form of a variable-period cosine function, uniform initialization, and a rank-based strategy for individual particle inertia weights. The cosine inertia weight introduced in this paper adopts the multistage strategy. It divides the change process of inertia weight into three stages. It can balance exploration and exploitation more specifically, help particles transform from global search to local search smoothly, and improve the convergence accuracy. Uniform initialization initializes a particle randomly as the basic point and then generates other initial particles based on this basic point. The initial particles are evenly distributed in each dimension, and the positions of each particle in each dimension are random. This mechanism can prevent the aggregation of initial particles. It distributes particles uniformly to recognize the search area more comprehensively. Uniform initialization is able to avoid falling into a local optimum and improve the search efficiency. In addition, this paper employs a rank-based strategy to adjust individual particle inertia weights. It makes the particles that are close to the swarm's best position focus on mining and makes particles that are far away from the swarm's best position keep exploring. It can enhance the global and local search ability of swarms at the same time.

In recent years, researchers have proposed some effective variants of PSO. Ye et al. proposed an improved multiswarm particle swarm optimization with dynamic learning strategy (PSO-DLS). It classified particles of each subswarm into ordinary particles and communication particles [25]. Lynn and Suganthan proposed the ensemble particle swarm optimizer (EPSO), which combines the characteristics of several PSO variants [26]. In EPSO, the best-performing algorithm for each generation can be determined by a self-adaptive scheme. Heterogeneous comprehensive learning particle swarm optimization (HCLPSO) divides the whole swarm into an exploration subpopulation and an exploitation subpopulation [27]. The CL strategy is used to breed learning exemplars for both of them. Gong et al. proposed genetic learning particle swarm optimization (GLPSO), which adopts selection, mutation, and selection [28]. By performing these operators on the historical information of particles, GLPSO is able to construct diversified and high-qualified learning exemplars to guide the swarm.

The purpose of designing UCPSO is to obtain a variant of PSO that has a good comprehensive performance and the ability to escape from a local optimum. In addition, three improvements in UCPSO should be easy to use. They are introduced to help researchers improve the global search ability and performance of PSO. A large number of comparative experiments based on benchmark functions were used to validate the effectiveness of the UCPSO improvements.

This paper is organized as follows.  Section 2 introduces the standard PSO and related research on inertia weight and particle initialization.  Section 3 describes the UCPSO and the three improvements in detail. Experiments are presented in Section 4. The conclusion is given in the Section 5.

## 2. Particle Swarm Optimization Algorithms

### 2.1. Standard PSO

**Figure 1:**
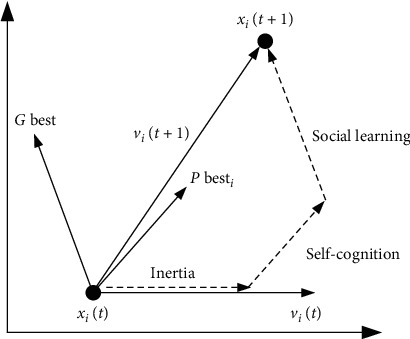
The concept of a particle's iteration.

PSO is a stochastic algorithm based on population [15]. It finds the optimal solution in a given range by mimicking the behaviour of birds. The particles in the swarm are potential solutions, and *n* is the total number of particles in the swarm. Every particle remembers its current position *X*_*i*_, its current velocity *V*_*i*_, and the best position that it has ever been *P*best_*i*_(1 ≤ *i* ≤ *n*). The swarm also remembers the swarm's best position *G*best. The *X*_*i*_, *V*_*i*_, *P*best_*i*_, *G*best are all *D*-dimensional vectors, *X*_*i*_=[*x*_*i*1_, *x*_*i*2_,…, *x*_*id*_,…, *x*_*iD*_], *V*_*i*_=[*v*_*i*1_, *v*_*i*2_,…*v*_*iD*_], *P*best_*i*_=[*p*best_*i*1_, *p*best_*i*2_,…, *p*best_*id*_,…, *p*best_*iD*_], *G*best=[*g*best_1_, *g*best_2_,…, *g*best_*d*_,…, *g*best_*D*_](1 ≤ *d* ≤ *D*). Particles find the optimal solution through iterations. The position and velocity of particles are updated as follows:

(1) $$\begin{array}{c} v_{id}\left(t+1\right)=\omega \times v_{id}\left(t\right)+c_{1}\times r_{1}\times \left[p{\text{best}}_{id}-x_{id}\left(t\right)\right]+c_{2}\times r_{2}\times \left[g{\text{best}}_{d}-x_{id}\left(t\right)\right], \end{array}$$

(2) $$\begin{array}{c} x_{id}\left(t+1\right)=x_{id}\left(t\right)+v_{id}\left(t+1\right), \end{array}$$

where *t* denotes the current iteration, *t* ≤ *t*_max_. In addition, *x*_*id*_ ∈ [*x*_*d*min_, *x*_*d*max_] and *v*_*i* *d*_ ∈ [*v*_*d*min_, *v*_*d*max_]. *ω* is the inertia weight. *c*1 and *c*2 are acceleration coefficients, which control the influence of *P*best_*i*_, and *G*best in the iteration. *r*1 and *r*2 are random numbers in the range 0, 1. PSO is usually terminated after reaching the allowed maximum number of iterations or meeting the stopping criterion. The best solution of a problem is the final *G*best.  Figure 1 shows the concept of a particle's iteration in a graphical way.

In equation (1), *ω* × *v*_*i* *d*_(*t*) represents the effect of inertia, *c*_1_ × *r*_1_ × [*p*best_*i* *d*_ − *x*_*i* *d*_(*t*)] represents the effect of self-cognition, and *c*_2_ × *r*_2_ × [*g*best_*d*_ − *x*_*i* *d*_(*t*)] represents the effect of social learning. The cooperation between them contributes to finding the optimal solution. The function used to evaluate the position of a particle is usually called the fitness function *F*(*X*_*i*_). To prevent particles from exceeding the search area, the position and velocity of a particle are always limited in the allowed range. When a particle reaches the boundary of the search area, its velocity should be reversed to improve the search efficiency. The pseudocode of the standard PSO is shown as follows (Algorithm 1):

### 2.2. Different Forms of Inertia Weight

The inertia weight reflects the influence of the previous velocity *V*_*i*_(*t*) on the new velocity *V*_*i*_(*t*+1). A large inertia weight can prevent particles from going to the region of interest (hereafter called the ROI) immediately. This makes particles continue to search outside the ROI for a period of time. A small inertia weight can make particles go to the ROI immediately and search in the ROI. That is, a large inertia weight enhances the global search capability (hereafter called exploration), and a small inertia weight enhances the local search capability (hereafter called exploitation). Exploration can prevent particles from falling into a local optimum, but it also leads to low convergence accuracy and a slow convergence speed. Exploitation can accelerate the convergence speed and improve the convergence accuracy, but it makes the algorithm converge prematurely or become trapped in a local optimum easily [29]. These two functions greatly influence the performance of PSO. Therefore, it is very important to choose an appropriate inertia weight.

Since inertia weight was proposed, many researchers have made contributions in this field. Some classical forms of inertia weight have been proposed, such as time invariant [15], linear time variant [16, 30], nonlinear time variant [17, 31], and other forms of inertia weight [32–34]. The famous forms of inertia weight mentioned above are described in detail in the subsections below.

#### 2.2.1. Time Invariant Inertia Weight

To improve the performance of the original PSO, Shi and Eberhart proposed a parameter called inertia weight *ω* to balance exploration and exploitation in 1998 [15]. First, the inertia weight appeared in the form of a constant. They found that a large inertia weight facilitates exploration, while a small inertia weight facilitates exploitation. The recommended range of inertia weight is [0.9, 1.2]. The computational results showed that the overall performance of PSO was improved empirically:

(3) $$\begin{array}{c} \omega_{0}\left(t\right)=\text{constant}, \end{array}$$

*ω*_0_(*t*) is easy to implement, so it has been widely used.

#### 2.2.2. Linear Time Variant Inertia Weight

After the concept of inertia weight was proposed, a linear time variant inertia weight was introduced in [16] to further improve the performance of the PSO algorithm. The mechanism of the linear time variant inertia weight *ω*_1_(*t*) is shown in the following equation:

(4) $$\begin{array}{c} \omega_{1}\left(t\right)=\omega_{\text{ini}}-\frac{t}{t_{\max}}\left(\omega_{\text{ini}}-\omega_{\text{fin}}\right),\;\omega_{\text{ini}}>\omega_{\text{fin}}. \end{array}$$

The initial value *ω*_ini_ to the final value *ω*_fin_ as the number of iterations increases. The linear time variant inertia weight takes the demands of particles in different periods into account. The recommended values of *ω*_ini_ and *ω*_fin_ are *ω*_ini_=1.4 and *ω*_fin_=0.

There are many other mechanisms of the linear time variant inertia weight. Specifically, Zheng proposed an increasing linear time variant inertia weight *ω*_2_(*t*)(*ω*_ini_ < *ω*_fin_). It is proven that the increasing mechanism performs better than the decreasing mechanism in some test functions [30]. The mechanism of inertia weight *ω*_2_(*t*) is

(5) $$\begin{array}{c} \omega_{2}\left(t\right)=\omega_{\text{ini}}+\frac{t}{t_{\max}}\left(\omega_{\text{fin}}-\omega_{\text{ini}}\right),\;\omega_{\text{ini}}<\omega_{\text{fin}}. \end{array}$$

#### 2.2.3. Nonlinear Time Variant Inertia Weight

Based on the linear time variant inertia weight, some researchers think that the nonlinear mechanism is more suitable for the demand of particles. Therefore, many nonlinear time variant mechanisms have been proposed. Chatterjee [31] introduced a nonlinear time variant inertia weight combined with a quadratic function, and its mechanism is

(6) $$\begin{array}{c} \omega_{3}\left(t\right)=\omega_{\text{ini}}+{\left(\frac{t}{t_{\max}}\right)}^{2}\left(\omega_{\text{ini}}-\omega_{\text{fin}}\right). \end{array}$$

For the sake of a better inertia weight, some researchers abandoned the continuous function and began to research the multiple stages of inertia weight. Tian [17] proposed a sigmoid increasing inertia weight *ω*_4_(*t*) and obtained an algorithm with satisfactory performance. The mechanism of inertia weight *ω*_4_(*t*) is as follows:

(7) $$\begin{array}{c} \omega_{4}\left(t\right)=\left\{\begin{array}{cc} \omega_{\text{ini}}, & t\leq 0.2\times t_{\max},\\ \frac{1}{\left(1+e^{\left(\left(10t-2t_{\max}\right)/t_{\max}\right)}\right)+\omega_{\text{fin}},} & \text{otherwise}. \end{array}\right. \end{array}$$

#### 2.2.4. Other Forms of Inertia Weight

Some researchers also proposed other effective strategies to adjust the inertia weight, such as random strategy, chaotic strategy, and self-adaptive strategy.

Randomness is the natural property of PSO, and it is also the reason why PSO can be applied to almost all optimization problems. It is difficult to predict whether exploration or exploitation would be better during the iteration. To address this problem, researchers thought of using random strategies to adjust the inertia weight. A random inertia weight *ω*_5_(*t*) was introduced in [32]. The mechanism of inertia weight *ω*_5_(*t*) is shown in

(8) $$\begin{array}{c} \omega_{5}\left(t\right)=0.5+\frac{\text{rand}\left(\right)}{2}, \end{array}$$

where rand() is a random number in the range [0, 1], so 0.5 ≤ *ω*_5_ ≤ 1.

Feng [33] used a chaotic strategy to adjust the inertia weight and obtain a chaotic inertia weight *ω*_6_(*t*). He added a chaotic term *z*(*t*) to the linearly decreasing inertia weight. The mechanism of inertia weight *ω*_6_(*t*) is

(9) $$\begin{array}{c} \omega_{6}\left(t\right)=\left(\omega_{\text{ini}}-\omega_{\text{fin}}\right)\frac{t_{\max}-t}{t_{\max}}+\omega_{\text{ini}}\times z\left(t\right), \end{array}$$

(10) $$\begin{array}{c} z\left(t+1\right)=4z\left(t\right)\left(1-z\left(t\right)\right), \end{array}$$

where the initial value of *z*(*t*) is a random number in the range [0, 1], and *z*(*t*) ≠ 0,0.25, 0.5, 0.75, 1.

Different from the random strategy and chaotic strategy, some researchers chose an index to adjust the value of inertia weight in real time. This kind of index can provide feedback on the state of the swarm. Zhang [34] et al. adopted an index *φ*i to monitor the state of each particle, and they proposed a self-adjusted inertia weight *ω*_7_(*t*). The mechanism of inertia weight *ω*_7_(*t*) is shown in the following equations:

(11) $$\begin{array}{c} \varphi_{i}\left(t+1\right)=\left|\frac{G\text{best}\left(t\right)-X_{i}\left(t\right)}{P{\text{best}}_{i}\left(t\right)-X_{i}\left(t\right)}\right|, \end{array}$$

(12) $$\begin{array}{c} \omega_{7}\left(t+1\right)=\frac{\omega_{\text{ini}}-\omega_{\text{fin}}}{1+e^{\varphi \left(t+1\right)\times \left(t-\left(\left(1+\ln\left(\varphi \left(t+1\right)\right)\right)\times t_{\max}\right)/\mu \right)}}+\omega_{\text{fin}}, \end{array}$$

where *μ* = 100. On the right side of equation (11), the numerator is the Euclidean distance from the position of the *i*^th^ particle to *G*best, and the denominator is the Euclidean distance from the position of the *i*^th^ particle to *P*best_*i*_. Therefore, the index *φ*_*i*_ can reflect the state of the *i*^th^ particle dynamically. The performance of the self-adaptive inertia weight in some test functions is excellent, but the mechanism of the self-adaptive inertia weight is usually very complicated. It is difficult to design a widely used index.

### 2.3. Particle Initialization

In addition, the quality of particle initialization is also critical to the performance of the PSO algorithm. The volatility of particle initialization is the primary cause of the volatility of convergence speed and accuracy.

#### 2.3.1. Random Initialization

The original method of particle initialization is random initialization. The position of each dimension of each particle is distributed in the allowed range independently and randomly, as shown in the following equation:

(13) $$\begin{array}{c} x_{i\;d}=\text{rand}\left(\right)\times \left(x_{d\max}-x_{d\min}\right)+x_{d\min}. \end{array}$$

After initialization, if particles are close to the global optimum, PSO tends to have good performance; if the particles are concentrated near the local optimum, PSO tends to fail. The random strategy of particle initialization inevitably leads to volatility of initialization. However, if particle initialization abandons randomness, it is very difficult for PSO to solve various optimization problems without prior knowledge. Fixed initialization can only solve specific problems.

#### 2.3.2. Chaotic Initialization

Chaotic initialization employs chaotic sequences to make particles more scattered. A common chaotic sequence called a logistic map is widely used because of its simple employment [35]. Its mechanism is as follows:

(14) $$\begin{array}{c} Z_{N}=a\times Z_{N-1}\times \left(1-Z_{N-1}\right), \end{array}$$

where *Z*_*N*_(1 ≤ *N* ≤ *n*) is the chaotic variable, *Z*_1_ is a random value in the range 0, 1, and other chaotic variables are obtained by equation (14). To prevent chaotic variables from falling into a cycle, *Z*_1_ ≠ 0, 0.25, 0.5, 0.75, and 1. *a* is a constant that controls the level of chaos, and the recommended range for a is [3.5699, 4]. In this paper, *Z*_*N*_ is obtained by equation (14) for 10 iterations to make the initial swarm more chaotic. After *n* × *D* chaotic variables are obtained, the chaotic initialization can be completed by replacing the random matrix with chaotic variables during the process of initialization.

#### 2.3.3. Opposition-Based Initialization

For two particles that are symmetrical about the centre of the search area, one particle of the two is closer to the global optimum than the other (it is a special case that the two distances are equal). Opposition-based initialization generates a subswarm randomly and combines it with its symmetric subswarm. Therefore, opposition-based initialization can avoid the situation in which all particles are far from the global optimum. Rahnamayan [21] introduced opposition-based initialization, and the mechanism of opposition-based initialization is shown in

(15) $$\begin{array}{c} x_{i\;d}=\left(x_{d\max}+x_{d\min}\right)-x_{\left(n+1-i\right)d} \end{array}$$

## 3. UCPSO Algorithm

Based on research on inertia weight and particle initialization, UCPSO is proposed to be a competitive variant of PSO. UCPSO adopts three new strategies, and their details are represented in the following sections.

### 3.1. Inertia Weight in the Form of Variable-Period Cosine Function

There is a popular form of nonlinear time variant inertia weight. It maintains a large value in the early stage and keeps a small value in the final stage. In common optimization problems, it can enhance the global search ability of PSO. Then, the multistage inertia weight was proposed. It puts forward more specific requirements for the change process of inertia weight: (a) initial stage: inertia weight keeps a large value for a period of time to carry out global search and reduces the probability of falling into local optimum (this stage is also called global search stage); (b) intermediate stage: inertia weight drops rapidly and transits from global search to local search (this stage is also called decelerating transition stage); (c) the final stage: inertia weight keeps a small value for a long time to help PSO converge to an accurate optimal solution quickly (this stage is also called the local search stage).

The change process of the cosine function in the range [0, *π*] meets the requirements of the multistage inertia weight. In the range [0, *π*/6], the cosine function maintains a large value $\left(\leq \sqrt{3}/2\right)$ and changes slowly. In the range [*π*/6, 5*π*/6], the cosine function declines rapidly. In the range [5*π*/6, *π*], the cosine function maintains a small value $\left(\leq -\sqrt{3}/2\right)$ and changes slowly. Because the cosine function is simple and easy to use, an inertia weight in cosine form is adopted in this paper. However, the original cosine function is not consistent with the requirements of the multistage inertia weight. Therefore, the original cosine function needs to be adjusted. An iterative term *I*(*t*) is added into the cosine function to adjust the period and *ω*_cos_(*t*) is rescaled in the range [*ω*_fin_, *ω*_ini_] as shown in the following equations:

(16) $$\begin{array}{c} \omega_{\cos}\left(t\right)=\frac{\left(\omega_{\text{ini}}+\omega_{\text{fin}}\right)}{2}+\frac{\left(\omega_{\text{ini}}-\omega_{\text{fin}}\right)}{2}\times \;\cos\left(\frac{I\left(t\right)\pi }{t_{\max}}\right), \end{array}$$

(17) $$\begin{array}{c} I\left(t+1\right)=I\left(t\right)+a,\;I\left(1\right)=0, \end{array}$$

(18) $$\begin{array}{c} a=\left\{\begin{array}{cc} a_{1}, & \frac{I\left(t\right)\leq t_{\max}}{6},\\ a_{2}, & \frac{t_{\max}}{6}<I\left(t\right)\leq \frac{5t_{\max}}{6},\\ a_{3}, & \frac{5t_{\max}}{6}<Ι\left(t\right)\leq t_{\max}, \end{array}\right. \end{array}$$

where *a* is a constant that can adjust the period of *ω*_cos_(*t*). The values of *a*_1_, *a*_2_, and *a*_3_ can control the length of each stage in *ω*_cos_(*t*) According to the requirements of *ω*_cos_(*t*), we limit the phase (*I*(*t*)*π*/*t*_max_) in the range 0, *π* and (*I*(*t*)*π*/*t*_max_) is required to increase from 0 to *π*. While *t* increases from 0 to *t*_max_*I*(*t*) needs to increase from 0 to *t*_max_. Therefore, *a*_1_, *a*_2_, and *a*_3_ have to satisfy the following equation:

(19) $$\begin{array}{c} \frac{1}{6a_{1}}+\frac{2}{3a_{2}}+\frac{1}{6a_{3}}=1. \end{array}$$

**Figure 2:**
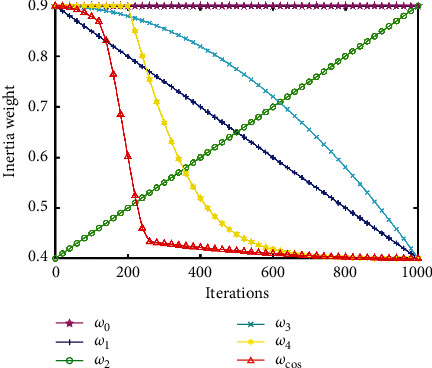
Curves of inertia weight *ω*_0_(*t*) ~ *ω*_4_(*t*) and *ω*_cos_(*t*).

**Figure 3:**
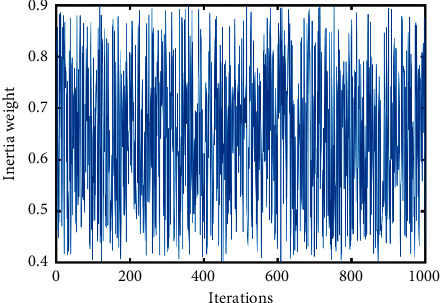
Curve of inertia weight *ω*_5_(*t*).

**Figure 4:**
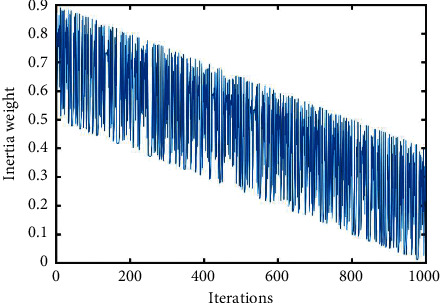
Curve of inertia weight *ω*_6_(*t*).

The pseudocode of updating *ω*_cos_ is shown as follows (Algorithm 2):

The parameter analysis experiment for *a*_1_, *a*_2_, and *a*_3_ is shown in Section 4.2. The recommended configuration of *a*_1_, *a*_2_,  and *a*_3_ is *a*_1_=(4/3), *a*_2_=(16/3),  and *a*_3_=(2/9). The curves of different inertia weights *ω*_0_(*t*) ~ *ω*_7_(*t*) and *ω*_cos_(*t*)(*a*_1_=(4/3), *a*_2_=(16/3), *a*_3_=(2/9)) are displayed in Figures 2–4. In addition, their parameters are also illustrated.

To make the inertia weight have the same value range, in these inertia weights, all *ω*_ini_ are set to 0.9, and all *ω*_fin_ are set to 0.4 (for *ω*_2_(*t*), *ω*_ini_=0.4, *ω*_fin_=0.9), *ω*_0_(*t*)=0.9,  and *ω*_5_(*t*)=0.4+rand()/2.

### 3.2. Uniform Initialization

There is no clear mechanism in random initialization, chaotic initialization, and opposition-based initialization to avoid the aggregation of initial particles. This situation will lead some areas to be searched repeatedly and some areas to be ignored. This reduces the search efficiency and the possibility of finding a global optimum.

**Figure 5:**
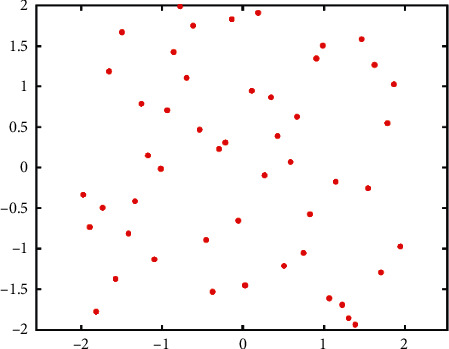
Results of the uniform initialization (2D).

To solve this problem, a particle initialization method with both randomness and uniformity (called uniform initialization) is proposed in this paper. In Algorithm 3, Line 1 initializes a particle randomly to be the base point, and *X*_1_=[*X*_11_, *X*_12_,…, *X*_1 *D*_] is the position of the base point. Lines 2–4 generate a *D* × (n − 1) random matrix *R*=[*R*_1_, *R*_2_,…, *R*_*D*_] to ensure the randomness of the initial particles. Lines 5–12 divide the length of each dimension by *n* to obtain the minimum distance between particles in the corresponding dimension. Lines 5–12 distribute particles in each dimension uniformly and avoid the aggregation of particles. If the position of a particle exceeds the allowed range of a dimension, it will subtract the range of the corresponding dimension. The pseudocode of uniform initialization is shown as follows:

Uniform initialization ensures that the distances between particles are larger than a certain value. It can avoid the aggregation of particles and distribute initial particles uniformly to recognize more areas at the beginning.  Figure 5 represents the result of uniform initialization. We find that the level of aggregation is low, and the distribution of particles is uniform. Uniform initialization is a good combination of randomness and uniformity.  Figure 5 is completed under the configuration: *n*=50, *D*=2, the search area is a 4 × 4 rectangle.

### 3.3. Rank-Based Strategy for Individual Particle's Inertia Weight (RIW)

The forms of inertia weight mentioned above are all assigned numerical values according to the state of the whole swarm. However, in fact, particles' states are diverse. The individual particle's need may not follow the swarm's need. Particles that are already in the ROI need a small inertia weight to exploit. Particles that are far away from the ROI need a large inertia weight to explore. The single value of inertia weight cannot satisfy both requirements at the same time. Cooperation between these two kinds of particles can maximize the benefit of the whole swarm.

A rank-based strategy is adopted by this paper to solve the problem. Generally, the particles with small fitness values (*F*(*X*_*i*_)) are in the current ROI, while the particles with large *F*(*X*_*i*_) are outside the ROI. Particles are sorted by fitness value from small to large. Adding a rank-based strategy to inertia weight can take both the overall and individual requirements into account simultaneously. The mechanism of the RIWs is shown in equations (20) and (21):

(20) $$\begin{array}{c} \omega_{i}=b_{i}\times \omega_{\text{swarm}}, \end{array}$$

(21) $$\begin{array}{c} b_{i}=\left\{\begin{array}{cc} b_{1}, & {\text{rank}}_{i}\leq \frac{n}{4},\\ b_{2}, & \text{otherwise,}\\ b_{3}, & {\text{rank}}_{i}\geq \frac{3n}{4}, \end{array}\right. \end{array}$$

where *ω*_*i*_ denotes the *i*^th^ particle's inertia weight, *ω*_swarm_ denotes the swarm's inertia weight, *b*_*i*_ is the adjustment factor for the *i*^th^ particle's inertia weight, and rank_*i*_ denotes the ranking of the *i*^th^ particle according to the fitness value.

The pseudocode of the RIWs is shown as follows (Algorithm 4):

The parameter analysis experiment for *b*_1_, *b*_3_ is shown in Section 4.2. The recommended configuration of *b*_1_, *b*_2_, *b*_3_ is *b*_1_=(2/3), *b*_2_=1, *b*_3_=1.5.

This paper adopts the above three mechanisms in the standard PSO and proposes a uniform initialized particle swarm optimization with cosine inertia weight (UCPSO). To elaborate the mechanism of UCPSO, the pseudocode of UCPSO is shown as follows (Algorithm 5):

## 4. Experimental Results and Discussion

### 4.1. Experimental Setup

The experiments in Sections 4.2–4.4 are performed on benchmark functions *f*_1_ − *f*_6_ [36]. The details of *f*_1_ − *f*_6_ are specified in Table 1. *f*_1_ − *f*_6_ include 2 many-local-minima functions, 2 bowl-shaped functions, and 2 valley-shaped functions. Therefore, *f*_1_ − *f*_6_ are representative. The experiment to compare UCPSO with PSO, MCJPSO [23], PSO-DLS [25], EPSO [26], HCLPSO [27], and GLPSO [28] and is based on CEC2020 benchmark functions, as shown in Table 2. The parameter configurations of the other six algorithms are set according to their original references, which are shown in Table 3.

The nonparametric Wilcoxon signed-rank test is used to examine the significant difference between algorithms. In this article, a Wilcoxon signed-rank test at a 5% significance level is used. A pairwise comparison is conducted over the results obtained through several runs. The symbol “+” indicates that the proposed algorithm performs significantly better than the compared algorithms. The symbol “=” indicates that the proposed algorithm is not significantly different from the compared algorithm. The symbol “−” indicates that the compared algorithm performs significantly better than the proposed algorithm.

The criteria include the mean of the best solutions (Mean), the standard deviation of the best solutions (SD), success rate (SR) [37] and the average number of iterations (Average number). SR reflects the probability of obtaining a satisfactory result. To reduce the impact of extreme values, only the successful iterations are counted when calculating average number. When the algorithm iterates successfully, the current number of iterations will be recorded to calculate average number, and the algorithm will continue to iterate. Average number reflects the convergence speed effectively when combined with SR. Whether the algorithm iterates successfully or unsuccessfully is judged according to the following content:

(22) $$\begin{array}{c} \varepsilon =\left\{\begin{array}{cc} 0.001, & F\left(X^{*}\right)=0,\\ 0.001\times \left|F\left(X^{*}\right)\right|, & F\left(X^{*}\right)\neq 0, \end{array}\right. \end{array}$$

where *ε* is the allowed maximum error. *ε* is often set as 0.001 in the engineering field. If |*F*(*G*best) − *F*(*X*^*∗*^)| ≤ *ε*, the current iteration is successful; if |*F*(*G*best) − *F*(*X*^*∗*^)| > *ε*, the current iteration is unsuccessful.

For a fair comparison, all algorithms use the following unified configuration: *n*=20, *t*_max_=2000, |*v*_*d*min_|=*v*_*d*max_=0.1 × (*x*_*d*max_ − *x*_*d*min_), and the initial velocity *v*_*i* *d*1_ is set to zero. For each test function, 1000 independent runs are performed in Sections 4.2–4.4, and 30 independent runs are performed in Section 4.5. The allowed maximum number of iterations is used as the termination criterion for all algorithms. All algorithms are implemented in MATLAB R2018b and executed on the same PC with an Intel® Core(TM) i7-7700 CPU @ 3.6 GHz and 16 GB RAM.

### 4.2. Parameter Analysis

Based on the standard PSO with *ω*_cos_(*t*), 15 different configurations of *a*_1_, *a*_2_, and *a*_3_ are compared on the benchmark functions *f*_1_∼*f*_6_. In these configurations, the duration of each stage changes in the step size of *t*_max_/8. Therefore, a relatively good parameter configuration can be obtained. All variants are tested on the experimental settings in Section 4.1.

In Table 4, when *a*_1_=4/3, *a*_2_=16/3, *a*_3_=2/9, the best performance is obtained. In Figure 2, we can see that the curve of *ω*_cos_(*t*) with *a*_1_=(4/3), *a*_2_=(16/3),  and *a*_3_=(2/9) satisfies the requirements of the multistage inertia weight.

The inertia weights of particles that are ranked in the middle remain constant, so the value of *b*_2_ is equal to 1. According to the requirements of the inertia weight of the particles, *b*_1_ < 1,  *b*_3_ > 1. Particles that are already in the ROI need a small inertia weight to exploit. Particles that are far away from the ROI need a large inertia weight to explore. Therefore, *b*_1_ should be less than 1 and *b*_3_ should be greater than 1. A minor adjustment of inertia weight enhances the cooperation between the swarm. If the inertia weight becomes too small, the diversity of the swarm will decrease. This situation will lead to premature convergence. If the inertia weight becomes too large, it will lead to low convergence accuracy because particles outside the ROI insufficiently participate in local search.

The effects of *b*_1_ and *b*_3_ are relatively independent. Therefore, the effects of different values of *b*_1_ or *b*_3_ on the standard PSO with RIWs are compared separately. The comparative experiments are based on benchmark functions *f*_1_ − *f*_6_. All variants are tested on the experimental settings in Section 4.1.

In Table 5, when *b*_1_=(2/3), the three best results are obtained. In Table 6, when *b*_3_=1.5, the three best results are obtained. When *b*_1_=2/3 and *b*_3_=1.5, the adjustment of inertia weight is not large. Therefore, the recommended parameters of RIWs are selected as *b*_1_=(2/3), *b*_2_=1,  and *b*_3_=1.5.

### 4.3. Comparing Inertia Weight *ω*_cos_(*t*) with Other Forms of Inertia Weight

To compare inertia weights *ω*_0_(*t*) ~ *ω*_7_(*t*) and *ω*_cos_(*t*), they are added into the standard PSO. The configurations of *ω*_0_(*t*) − *ω*_6_(*t*) and *ω*_cos_(*t*) are elaborated in Section 3.1. In the inertia weight *ω*_7_(*t*), *ω*_ini_=0.9 and *ω*_fin_=0.4. For brevity, the standard PSO with inertia weight *ω*_0_(*t*) is abbreviated as PSO-*ω*_0_ and so on. All variants are tested on the experimental settings in Section 4.1.

Table 7 shows the performance of standard PSO variants with nine different forms of inertia weight. If the *SR* of a variant is 0, its average number will be omitted. We can find that PSO-*ω*_cos_ has the highest *SR* in *f*_1_ − *f*_5_. In *f*_6_ PSO-*ω*_cos_ still obtains the second highest SR. *ω*_cos_(*t*) maintains a large value for a period of time, so its global search ability is improved. PSO-*ω*_*cos*_ has the smallest mean of the four benchmark functions and the smallest SD of the three benchmark functions. In *f*_5_ and *f*_6_, PSO-*ω*_2_ has the smallest mean and SD, but its SR is still lower than PSO-*ω*_cos_‘s *SR*. *ω*_cos_(*t*) adopted a multistage strategy to meticulously guide the behaviour of particles at different stages. Therefore, PSO-*ω*_cos_ obtains a better convergence quality than other variants. PSO-*ω*_cos_ has the fastest convergence speed in *f*_5_ and a moderate convergence speed in the other five benchmark functions.

**Figure 6:**
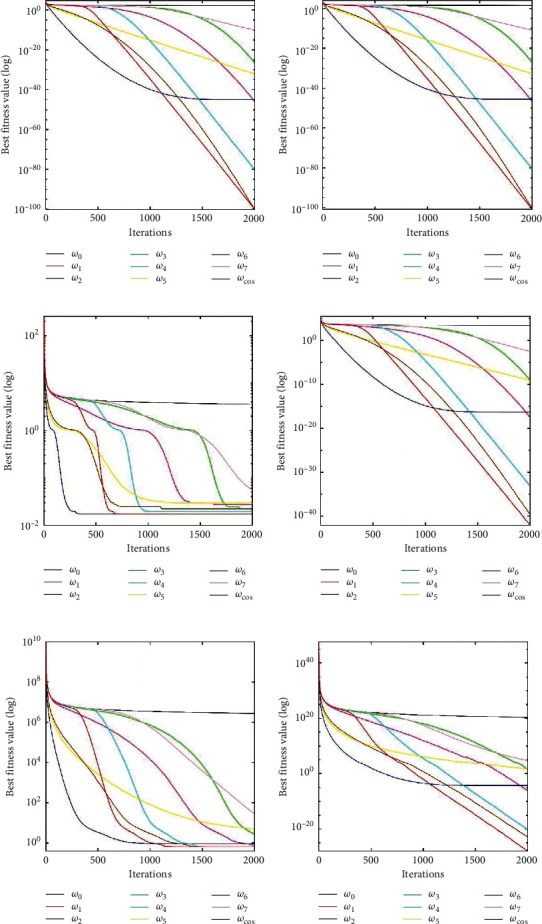
The median convergence curves of the standard PSO with inertia weight *ω*_0_(*t*) ~ *ω*_7_(*t*) and *ω*_*cos*_(*t*) on benchmark functions *f*_1_ ~  *f*_6_. (a) *f*_1_. (b) *f*_2_. (c) *f*_3_. (d) *f*_4_. (e) *f*_5_. (f) *f*_6_.

Figure 6 shows the convergence characteristics of the nine variants. The convergence speed of PSO-*ω*_cos_ is not very fast at the beginning, but PSO-*ω*_cos_ converges to the smallest fitness value in all six benchmark functions. In *f*_4_ and *f*_6_, PSO-*ω*_cos_ outperforms the other variants. PSO-*ω*_cos_ is able to avoid becoming trapped in the local optimum *ω*_cos_(*t*) converts to local search quickly and smoothly. These factors help PSO-*ω*_cos_ converge to an accurate solution.

### 4.4. Comparing Uniform Initialization with Other Particles Initializations

Random initialization, chaotic initialization, opposition-based initialization, and uniform initialization are compared on the benchmark functions *f*_1_ − *f*_6_. The four particle initializations are all added to the standard PSO-*ω*_1_ to be compared. The configurations of random initialization, chaotic initialization and opposition-based initialization are elaborated in Section 2.3. For chaotic initialization, *a* is set to 4. Uniform initialization adopts the recommended configuration in Section 3.2. The four variants are abbreviated as PSO-rand, PSO-chaotic, PSO-opposition, and PSO-uniform for simplicity. All variants are tested on the experimental settings in Section 4.1.

From Table 8, it can be seen that PSO-uniform performs better than the compared variants. PSO-uniform obtains the smallest mean in five benchmark functions and the highest SR in four benchmark functions. If the initial particles gather around a local optimum, the iteration is very likely to converge prematurely. Uniform initialization reduces the possibility of this case. It distributes initial particles more uniformly and makes them more likely to approach the global optimum. This improves the SR and convergence quality of the PSO algorithm. Except for *f*_5_, PSO-uniform has at least the second smallest average number. In addition, it has the smallest Average number in *f*_1_. It can be concluded that PSO-uniform has a better global search ability and a better convergence speed in *f*_1_ − *f*_6_.

### 4.5. Comparing UCPSO with Other Variants of PSO

To analyse the performance of UCPSO, UCPSO is compared with PSO, MCJPSO [23], PSO-DLS [25], EPSO [26], HCLPSO [27], and GLPSO [28] on the CEC2020 benchmark functions. The problem dimension of the experiment is set to *D*=20. UCPSO adopts the following configuration: *a*_1_=(4/3), *a*_2_=(16/3), *a*_3_=(2/9), *b*_1_=(2/3), *b*_2_=1, *b*_3_=1.5, *ω*_ini_=0.9, and *ω*_fin_=0.4. All algorithms are tested on the experimental settings in Section 4.1. For each test function, 30 independent runs are performed.

From Table 9, it can be observed that UCPSO outperforms PSO and PSO-DLS in 20-dimensional CEC2020 benchmark functions. Except for *F*_*1*_, *F*_8_, and *F*_9_, UCPSO has better results than MCJPSO. This indicates that the performance of PSO is enhanced by the proposed three improvements. HCLPSO achieves an excellent result in this experiment. In most benchmark functions, UCPSO can keep up with HCLPSO. In *F*_4_ and *F*_10_, UCPSO performs almost as well as HCLPSO. In *F*_5_, UCPSO achieves the best result. In hybrid and composition functions, UCPSO obtains good results and outperforms GLPSO. This proves that the performance of UCPSO is very competitive and that the proposed three improvements are effective.

**Figure 7:**
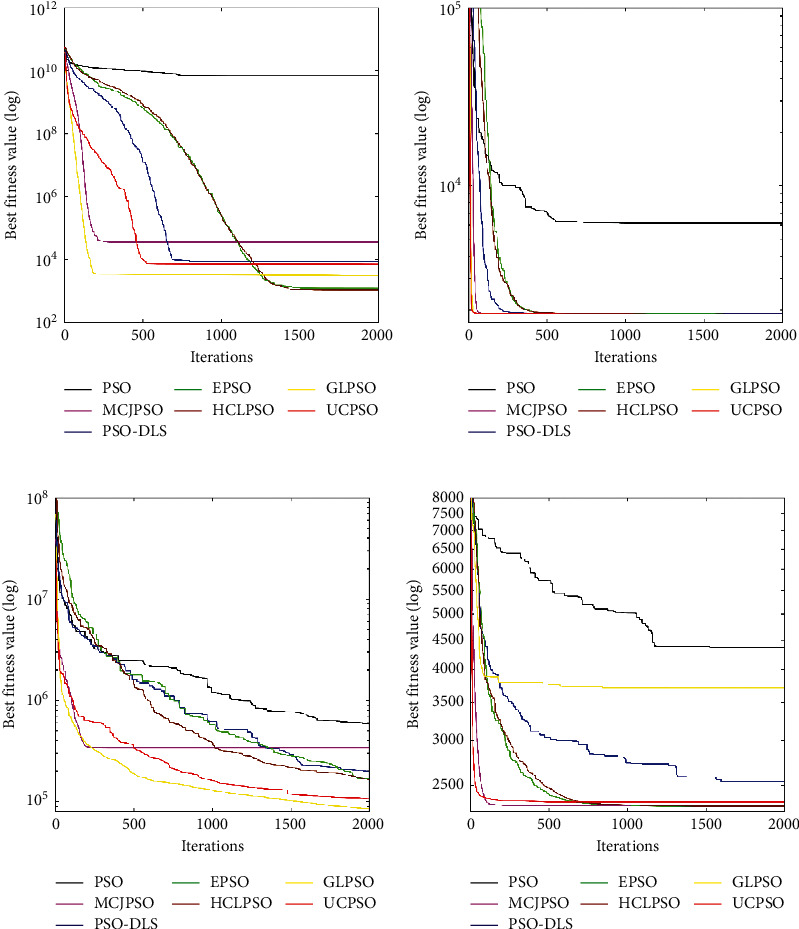
The median convergence curves on 20-Dimensional CEC2020 benchmark functions (a) *F*_1_. (b) *F*_4_. (c) *F*_5_. (d) *F*_8_.

In Figure 7, the median convergence curves of the four types of benchmark functions are shown. We can see that UCPSO has good convergence performance, especially in the top 500 iterations. UCPSO converges fast and has a high level of convergence accuracy, especially in *F*_4_ and *F*_5_. UCPSO can maintain a strong exploration and exploitation ability and converge to an accurate solution in a short time.

### 4.6. Algorithm Complexity

This section will analyse the computational complexity of UCPSO. The computational cost of the original PSO involves the initialization (*T*_ini_), evaluation (*T*_eva_), velocity, and position update (*T*_upd_) for each particle. *D* is the dimensionality of the search space, and *t*_max_ is the allowed maximum number of iterations. The computational complexity of PSO can be estimated as *T*(*D*)=*T*_ini_+(*T*_eva_+*T*_upd_)*t*_max_=*D*+(*D*+2 · *D*) *t*_max_=*D*(1+3*t*_max_). Therefore, the computational complexity of the original PSO is *O*(*D* · *t*_max_)*ω*_cos_(*t*) can be calculated in advance, so it is used directly during the iteration process. Uniform initialization adds a process of sorting before the iteration begins. RIWs adds the process of sorting and assignment into each iteration. Therefore, the computational complexity of UCPSO can be estimated as follows: *T*(*D*)=*T*_ini_+(*T*_eva_+*T*_upd_)*t*_max_=(*D*+*D* ·  log(*n*))+(*D*+2 · *D* +log(*n*)+*D*)*t*_max_=*D*(1+log(*n*)+4*t*_max_)+log(*n*) *t*_max_. Because the number of particles *n* is usually small, the computational complexity of UCPSO is *O*(*D* · *t*_max_)too.

An experiment to compare the computational complexity of UCPSO with other PSO variants is carried out. *T*_0_ is the time to run the following codes:

(23) $$\begin{array}{c} \text{For }i=1:200000,\\ x=\text{i}+5.5;\\ x=x+x;\\ x=\frac{x}{2},\\ x=x*x;\\ x=\text{sqrt}\left(x\right);\\ x=\text{log}\left(x\right);\\ \text{x}=\text{exp}\left(x\right);\\ y=\frac{x}{x};\\ \text{End}. \end{array}$$

where *T*_1_ is the time to execute 40,000 evaluations of benchmark function *F*_1_ by itself with 20 dimensions and *T*_2_ is the mean time to execute the algorithm with 40,000 evaluations of *F*_1_ with 20 dimensions over 30 times. The number of particles is 20.

According to Table 10, UCPSO spends the least time on *F*_1_ apart from PSO. PSO-DLS is up to 2 times slower in terms of time than UCPSO. The computational complexity of UCPSO is lower than those of PSO-DLS, EPSO, and HCLPSO. Therefore, UCPSO is a relatively fast PSO algorithm with competitive performance. The three improvements in UCPSO will not greatly increase the computational complexity.

### 4.7. Application to Real-World Problems

In this part, UCPSO is applied to solve real-world engineering optimization problems. PSO and UCPSO are tested on *P*_1_ and *P*_4_ of the CEC2011 real-world optimization problems, as shown in Table 11. For each test problem, 30 independent runs are performed. The population size is set to 20, and the maximum number of iterations is set to 2000. The allowed maximum number of iterations is used as the termination criterion for all algorithms.

#### 4.7.1. Parameter Estimation for Frequency-Modulated (FM) Sound Waves

Frequency-modulated (FM) sound wave synthesis has an important role in several modern music systems [38]. The object of this problem is to minimize the summation of square errors between the following equations:

(24) $$\begin{array}{c} y\left(\text{t}\right)=a_{1}\sin\left(\omega_{1}\cdot t\cdot \theta -a_{2}\cdot \;\sin\left(\omega_{2}\cdot t\cdot \theta +a_{3}\cdot \;\sin\left(\omega_{3}\cdot t\cdot \theta \right)\right)\right), \end{array}$$

(25) $$\begin{array}{c} y_{0}\left(\text{t}\right)=1.0\;\sin\left(5.0\cdot t\cdot \theta -1.5\cdot \;\sin\left(4.8\cdot t\cdot \theta +2.0\cdot \;\sin\left(4.9\cdot t\cdot \theta \right)\right)\right), \end{array}$$

where *θ*=(2*π*/100). The dimension of this problem is 6, and the search range is [−6.4, 6.35]. The fitness function is shown as

(26) $$\begin{array}{c} \begin{array}{c} f\left(x\right)=\overset{100}{\underset{t=0}{\sum }}{\left(y\left(t\right)-y_{0}\left(t\right)\right)}^{2} \end{array}. \end{array}$$

#### 4.7.2. Optimal Control of a Nonlinear Stirred Tank Reactor

This problem is a multimodal optimal control problem. It describes a first-order irreversible chemical reaction carried out in a continuous stirred tank reactor [38]. This chemical process is modelled by two nonlinear differential equations:

(27) $$\begin{array}{c} {\dot{x}}_{1}=-\left(2+u\right)\left(x_{1}+0.25\right)+\left(x_{2}+0.5\right)\exp\left(\frac{25x_{1}}{\left(x_{1}+2\right)}\right),\\ \begin{array}{c} {\dot{x}}_{2}=0.5-x_{2}-\left(x_{2}+0.5\right)\exp\left(\frac{25x_{1}}{\left(x_{1}+2\right)}\right), \end{array} \end{array}$$

where *u* is the flow rate of the cooling fluid, *x*_1_ is the dimensionless steady-state temperature, and *x*_2_ is the deviation from the dimensionless steady-state concentration. The fitness function of this problem is

(28) $$\begin{array}{c} \begin{array}{c} J=\int_{0}^{t_{f}=0.72}\left(x_{1}^{2}+x_{2}^{2}+0.1u^{2}\right)\text{d}t \end{array}. \end{array}$$

The initial condition is *x*_1_=*x*_2_=0.09. The search range is unconstrained, but the initial range of *u* is [0,5]. The dimension of this problem is 1.

As shown in Table 12, the best result of UCPSO is slightly worse than the best result of PSO, but the worst result of UCPSO is better than the worst result of PSO. In Table 13, the best and worst results of UCPSO are better than the best and worst results of PSO, respectively. According to the results on *P*_1_ and *P*_4_, UCPSO has the ability to solve real-world engineering optimization problems.

## 5. Conclusion

In this paper, UCPSO is proposed to prevent PSO from falling into a local optimum and improve the comprehensive performance of PSO. It adopts a variable-period cosine inertia weight *ω*_cos_(*t*), uniform initialization, and rank-based strategy for individual particle inertia weights (RIWs). *ω*_cos_(*t*) can satisfy the requirements of inertia weight at different stages and balance exploration and exploitation better. Uniform initialization is able to avoid the aggregation of initial particles. RIWs increase the diversity of swarms and improves exploration and exploitation at the same time. The above three improvements enhance the global search ability of PSO and ensure a competitive comprehensive performance of UCPSO. Extensive tests based on benchmark functions validate the effectiveness of the improvements and the performance of UCPSO.

In future work, the authors will perform more experiments to obtain a better configuration of parameters. We intend to apply UCPSO to practical engineering fields, such as clustering, parameter optimization, image segmentation, and industry scheduling. After that, we will continue to study other forms of inertia weight and research more effective improvements that are easy to implement.

## Figures and Tables

**Algorithm 1:**
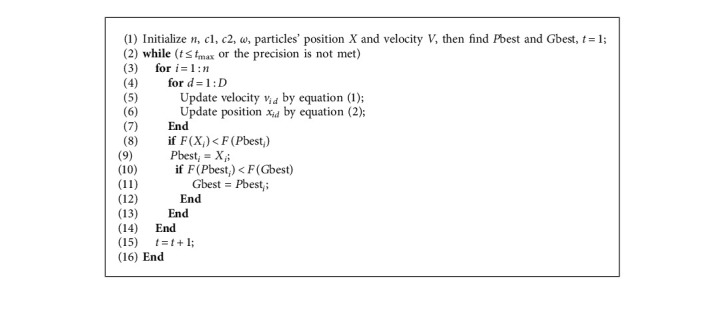
Pseudocode of the standard PSO.

**Algorithm 2:**
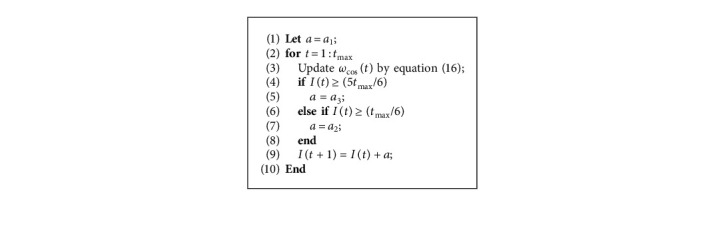
Pseudocode of updating *ω*_cos_(*t*).

**Algorithm 3:**
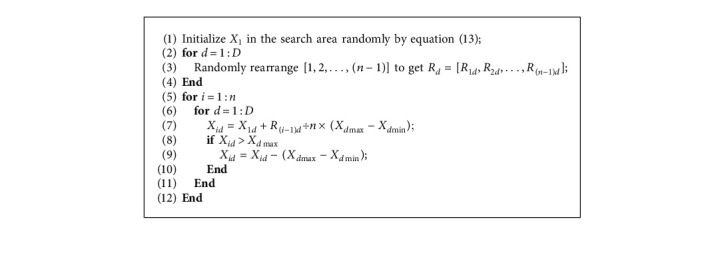
Pseudocode of uniform initialization.

**Algorithm 4:**
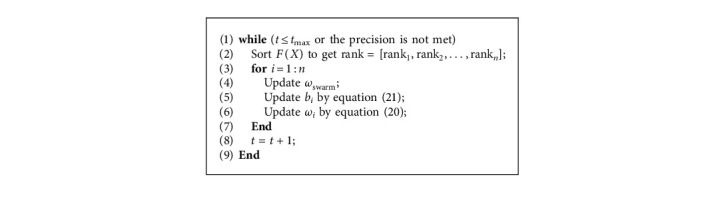
Pseudocode of RIWs.

**Algorithm 5:**
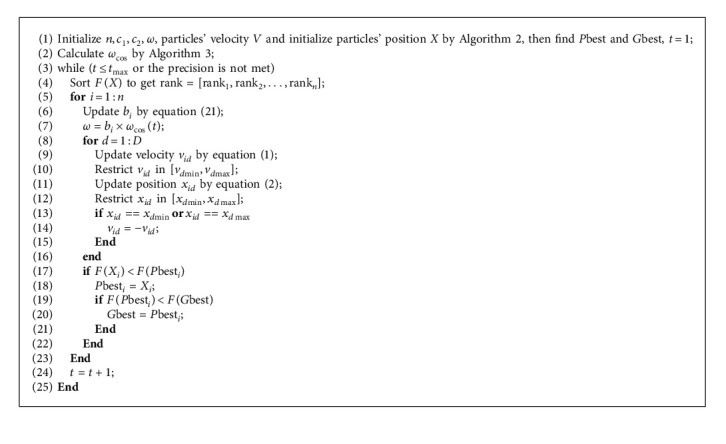
Pseudocode of UCPSO.

**Table 1:** Commonly used benchmark functions *f*_1_∼*f*_6_.

Benchmark function	Expression	*D*	Search range	*F*(*X*^*∗*^)
Rotated hyper-ellipsoid (10)	*f*_1_(*x*)=∑_*i*=1_^10^∑_*j*=1_^*i*^*x*_*j*_^2^	10	[−100, 100]^10^	0
Sphere (10)	*f*_2_(*x*)=∑_*i*=1_^10^*x*_*i*_^2^	10	[−100, 100]^10^	0
Griewank (20)	$f_{6}\left(x\right)=\sum_{i=1}^{20}\left(x_{i}^{2}/4000\right)-\prod_{i=1}^{20}\cos\left(x_{i}/\sqrt{i}\right)+1$	20	[−600, 600]^20^	0
Sum squares (20)	*f*_4_(*x*)=∑_*i*=1_^20^*ix*_*i*_^2^	20	[−100, 100]^20^	0
Dixon-price (30)	*f*_5_(*x*)=(*x*_1_ − 1)^2^+∑_*i*=2_^30^*i*(2*x*_*i*_^2^ − *x*_*i*−1_)^2^	30	[−100, 100]^30^	0
Sum of different powers (30)	*f*_6_(*x*)=∑_*i*=1_^30^|*x*_*i*_|^*i*+1^	30	[−100, 100]^30^	0

**Table 2:** CEC2020 benchmark functions.

Type	No.	Functions	Search range
Unimodal	*F*1	Shifted and rotated bent cigar function	[−100, 100]^D^

Basic	*F*2	Shifted and rotated Schwefel's function	[−100, 100]^D^
	*F*3	Shifted and rotated lunacek bi-Rastrigin function	[−100, 100]^D^
	*F*4	Expanded Rosenbrock's plus Griewank's function	[−100, 100]^D^

Hybrid	*F*5	Hybrid function 1 (*N* = 3)	[−100, 100]^D^
	*F*6	Hybrid function 2 (*N* = 4)	[−100, 100]^D^
	*F*7	Hybrid function 3 (*N* = 5)	[−100, 100]^D^

Composition	*F*8	Composition function 1 (*N* = 3)	[−100, 100]^D^
	*F*9	Composition function 2 (*N* = 4)	[−100, 100]^D^
	*F*10	Composition function 3 (*N* = 5)	[−100, 100]^D^

**Table 3:** Parameters settings for the involved algorithms.

Algorithms	Parameter settings	Reference
PSO	*ω* : 0.9∼0.4, *c*_1_ = *c*_2_ = 2	[15]
MCJPSO	*ω* : 1∼0, *c*_1_ : 1.5∼0, *c*_2_ : 0∼1.5, *c*_3_ : 1.5∼0, *k* = 5, *α* = 30, *ε* = 0.0001	[23]
PSO-DLS	*ω* : 0.9∼0.4, *c*_1_ = *c*_2_ = 1.49445, *M* = 4	[25]
EPSO	PSO: *ω* : 0.9∼0.2, *c*_1_ : 2.5∼0.5, *c*_2_ : 0.5∼2.5 LIPS: 𝜒 = 0.729, *c* = 2, *n*size = 3 FDR-PSO: *ω* : 0.9∼0.2, 𝜒 = 0.729, *c*_1_ = *c*_2_ = 1, *c*_3_ = 2 CLPSO: *ω* : 0.9∼0.2, *c* : 3–1.5 sHPSO: *ω* = 0.72, *c*_1_ : 2.5∼0.5, *c*_2_ : 0.5∼2.5	[26]
HCLPSO	*ω* : 0.99∼0.2, *c*_1_ : 2.5∼0.5, *c*_2_ = 0.5∼2.5, *c* : 3∼1.5	[27]
GLPSO	*ω* = 0.7298, *c* = 1.49618, pm = 0.01, sg = 7	[28]

**Table 4:** Performance of PSO with different *a*_1_, *a*_2_, and *a*_3_.

(*a*_1_, *a*_2_, *a*_3_)	*f*_1_	*f*_2_	*f*_3_	*f*_4_	*f*_5_	*f*_6_
(4/3, 8/9, 4/3)	2.456*E* − 47	4.123*E* − 47	3.035*E* − 02	2.533*E* − 18	2.706*E* + 02	1.010*E* + 29
(4/3, 16/15, 2/3)	9.234*E* − 57	1.880*E* − 57	2.884*E* − 02	5.596*E* − 23	3.315*E* + 02	2.000*E* + 31
(4/3, 4/3, 4/9)	1.125*E* − 65	4.724*E* − 67	2.720*E* − 02	2.273*E* − 27	2.604*E* + 02	3.051*E* + 25
(4/3, 16/9, 1/3)	3.091*E* − 76	9.313*E* − 77	2.743*E* − 02	2.000*E* + 01	2.907*E* + 02	1.011*E* + 25
(4/3, 8/3, 4/15)	6.126*E* − 86	4.224*E* − 86	2.563*E* − 02	3.611*E* − 36	4.825*E* + 02	2.041*E* + 27
(4/3, 16/3, 2/9)	**2.466*E* − 96**	**2.674*E* − 95**	2.397*E* − 02	**7.000*E* − 40**	2.111*E* + 02	2.010*E* + 27
(2/3, 16/15, 4/3)	8.895*E* − 42	5.300*E* − 42	2.878*E* − 02	2.078*E* − 16	3.607*E* + 02	1.011*E* + 29
(2/3, 4/3, 2/3)	4.436*E* − 50	1.295*E* − 51	2.847*E* − 02	1.623*E* − 20	4.508*E* + 02	1.031*E* + 25
(2/3, 16/9, 4/9)	3.922*E* − 61	2.144*E* − 61	2.672*E* − 02	6.873*E* − 25	**1.900*E* + 02**	2.010*E* + 27
(2/3, 8/3, 1/3)	3.936*E* − 70	8.225*E* − 71	2.411*E* − 02	6.847*E* − 29	2.215*E* + 02	1.010*E* + 29
(2/3, 16/3, 4/15)	4.251*E* − 81	6.035*E* − 80	2.447*E* − 02	4.645*E* − 33	2.604*E* + 02	**4.101*E* + 23**
(4/9, 4/3, 4/3)	1.855*E* − 35	9.454*E* − 37	2.865*E* − 02	3.827*E* − 14	2.522*E* + 02	2.010*E* + 29
(4/9, 16/9, 2/3)	1.404*E* − 45	2.728*E* − 46	2.577*E* − 02	2.206*E* − 18	4.120*E* + 02	2.030*E* + 27
(4/9, 8/3, 4/9)	3.625*E* − 55	8.133*E* − 56	2.413*E* − 02	9.497*E* − 23	2.509*E* + 02	1.001*E* + 29
(4/9, 16/3, 1/3)	3.834*E* − 65	3.807*E* − 65	**2.330*E* − 02**	2.433*E* − 27	3.512*E* + 02	1.000*E* + 31

The best mean of fitness value is shown in bold.

**Table 5:** Performance of PSO with different *b*_1_.

*b*_1_	*f*_1_	*f*_2_	*f*_3_	*f*_4_	*f*_5_	*f*_6_
2/3	**7.364*E* − 65**	**2.737*E* − 65**	**4.243*E* − 02**	9.559*E* − 28	1.807*E* + 02	1.000*E* + 31
½	1.360*E* − 64	5.581*E* − 65	6.441*E* − 02	**1.114*E* − 28**	1.198*E* + 02	1.000*E* + 29
2/5	1.692*E* − 63	1.464*E* − 64	7.144*E* − 02	1.618*E* − 28	1.984*E* + 02	1.000*E* + 27
1/3	3.013*E* − 64	1.470*E* − 64	5.802*E* − 02	3.746*E* − 28	2.286*E* + 02	**1.030*E* + 25**
2/7	1.061*E* − 63	2.657*E* − 63	5.469*E* − 02	1.588*E* − 27	1.696*E* + 02	2.020*E* + 25
¼	1.296*E* − 62	2.972*E* − 64	5.336*E* − 02	6.460*E* − 28	1.305*E* + 02	1.010*E* + 27
2/9	4.161*E* − 63	1.133*E* − 63	5.252*E* − 02	1.327*E* − 27	1.192*E* + 02	2.010*E* + 25
1/5	5.382*E* − 63	1.012*E* − 62	5.041*E* − 02	1.996*E* − 26	**6.069*E* + 01**	1.000*E* + 27

The best mean of fitness value is shown in bold.

**Table 6:** Performance of PSO with different *b*_3_.

*b*_3_	*f*_1_	*f*_2_	*f*_3_	*f*_4_	*f*_5_	*f*_6_
1.5	4.099*E* − 40	**3.495*E* − 41**	3.277*E* − 02	**2.894*E* − 15**	**4.328*E* + 02**	2.010*E* + 29
2	6.318*E* − 40	6.602*E* − 41	3.169*E* − 02	4.699*E* − 15	4.623*E* + 02	4.040*E* + 27
2.5	4.138*E* − 40	1.639*E* − 40	3.209*E* − 02	4.261*E* − 15	6.133*E* + 02	1.000*E* + 31
3	1.355*E* − 40	1.940*E* − 40	**3.078*E* − 02**	2.000*E* + 01	5.915*E* + 02	1.020*E* + 29
3.5	3.958*E* − 40	5.518*E* − 41	3.165*E* − 02	4.699*E* − 15	5.639*E* + 02	1.010*E* + 31
4	2.140*E* − 40	4.549*E* − 41	3.364*E* − 02	3.311*E* − 15	5.128*E* + 02	**1.041*E* + 27**
4.5	4.637*E* − 40	4.830*E* − 41	3.131*E* − 02	4.246*E* − 15	6.023*E* + 02	2.041*E* + 27
5	**7.087*E* − 41**	6.363*E* − 41	3.340*E* − 02	2.908*E* − 15	7.840*E* + 02	1.000*E* + 33

The best mean of fitness value is shown in bold.

**Table 7:** Results of the experiment on benchmark functions *f*_1_ ~ *f*_6_.

Benchmark function	Variant	Mean	SD	SR (%)	Average number
*f*_1_	PSO-*ω*_0_	1.433*E* + 02(+)	4.434*E* + 01	0	—
	PSO-*ω*_1_	1.266*E* − 43(+)	2.313*E* − 42	**100**	9.702*E* + 02
	PSO-*ω*_2_	3.008*E* − 43(+)	2.350*E* − 42	**100**	**9.732*E* + 01**
	PSO-*ω*_3_	1.924*E* − 25(+)	2.446*E* − 24	**100**	1.436*E* + 03
	PSO-*ω*_4_	3.686*E* − 76(+)	6.118*E* − 75	**100**	7.431*E* + 02
	PSO-*ω*_5_	8.088*E* − 31(+)	4.899*E* − 30	**100**	2.950*E* + 02
	PSO-*ω*_6_	6.636*E* − 95(+)	1.041*E* − 93	**100**	3.195*E* + 02
	PSO-*ω*_7_	2.146*E* − 10(+)	4.798*E* − 10	**100**	1.443*E* + 03
	PSO-*ω*_*cos*_	**3.471*E*** **−** **96**	**7.484*E* − 95**	**100**	4.878*E* + 02

*f*_2_	PSO-*ω*_0_	3.265*E* + 01(+)	9.727*E* + 00	0	—
	PSO-*ω*_1_	1.322*E* − 43(+)	3.828*E* − 42	**100**	9.298*E* + 02
	PSO-*ω*_2_	1.420*E* − 43(+)	2.048*E* − 42	**100**	**8.715*E* + 01**
	PSO-*ω*_3_	5.474*E* − 26(+)	6.638*E* − 25	**100**	1.403*E* + 03
	PSO-*ω*_4_	2.540*E* − 76(+)	6.720*E* − 75	**100**	7.250*E* + 02
	PSO-*ω*_5_	2.549*E* − 31(+)	1.781*E* − 30	**100**	2.555*E* + 02
	PSO-*ω*_6_	2.688*E* − 95(+)	4.673*E* − 94	**100**	2.897*E* + 02
	PSO-*ω*_7_	2.774*E* − 11(+)	4.776*E* − 11	**100**	1.382*E* + 03
	PSO-*ω*_*cos*_	**8.009*E*** − **97**	**9.211*E* − 96**	**100**	4.759*E* + 02

*f*_3_	PSO-*ω*_0_	3.524*E* + 00(+)	4.407*E* − 01	0	—
	PSO-*ω*_1_	3.281*E* − 02(+)	2.894*E* − 02	13.4	1.372*E* + 03
	PSO-*ω*_2_	2.582*E* − 02(+)	2.629*E* − 02	19.7	**2.413*E* + 02**
	PSO-*ω*_3_	3.306*E* − 02(+)	3.054*E* − 02	12	1.738*E* + 03
	PSO-*ω*_4_	2.494*E* − 02(+)	**2.377*E* − 02**	17.8	9.392*E* + 02
	PSO-*ω*_5_	3.599*E* − 02(+)	4.522*E* − 02	12.7	9.810*E* + 02
	PSO-*ω*_6_	2.825*E* − 02(+)	2.657*E* − 02	15.9	6.970*E* + 02
	PSO-*ω*_7_	7.983*E* − 02(+)	7.891*E* − 02	1.7	1.950*E* + 03
	PSO-*ω*_*cos*_	**2.217*E*** − **02**	2.520*E* − 02	**22.8**	6.320*E* + 02

*f*_4_	PSO-*ω*_0_	2.626*E* + 03(+)	4.838*E* + 02	0	—
	PSO-*ω*_1_	4.126*E* − 17(+)	2.101*E* − 16	**100**	1.356*E* + 03
	PSO-*ω*_2_	1.092*E* − 15(+)	1.001*E* − 14	**100**	**2.571*E* + 02**
	PSO-*ω*_3_	4.221*E* − 09(+)	1.786*E* − 08	**100**	1.749*E* + 03
	PSO-*ω*_4_	9.650*E* − 32(+)	1.930*E* − 30	**100**	9.382*E* + 02
	PSO-*ω*_5_	5.235*E* − 09(+)	2.027*E* − 08	**100**	9.729*E* + 02
	PSO-*ω*_6_	7.056*E* − 38(+)	1.150*E* − 36	**100**	6.657*E* + 02
	PSO-*ω*_7_	5.250*E* − 03(+)	3.345*E* − 02	9.2	1.976*E* + 03
	PSO-*ω*_*cos*_	**6.008*E*** − **40**	**9.875*E* − 39**	**100**	6.351*E* + 02

*f*_5_	PSO-*ω*_0_	2.787*E* + 06(+)	7.504*E* + 05	0	—
	PSO-*ω*_1_	2.106*E* + 02(−)	2.115*E* + 03	0	—
	PSO-*ω*_2_	**1.456*E*** **+** **00**(−)	**1.213*E* + 00**	0	—
	PSO-*ω*_3_	1.797*E* + 02(−)	2.037*E* + 03	0	—
	PSO-*ω*_4_	3.315*E* + 02(+)	2.841*E* + 03	0.9	1.756*E* + 03
	PSO-*ω*_5_	1.065*E* + 02(−)	1.680*E* + 03	0	—
	PSO-*ω*_6_	1.204*E* + 02(−)	1.548*E* + 03	0.8	1.514*E* + 03
	PSO-*ω*_7_	5.127*E* + 02(+)	3.494*E* + 03	0	—
	PSO-*ω*_*cos*_	2.410*E* + 02	2.449*E* + 03	**1.2**	**1.438*E* + 03**

*f*_6_	PSO-*ω*_0_	2.031*E* + 25(+)	4.468*E* + 26	0	—
	PSO-*ω*_1_	1.000*E* + 29(+)	3.161*E* + 30	65.4	1.810*E* + 03
	PSO-*ω*_2_	**5.031*E*** + **13**(−)	**7.054*E* + 14**	72.2	**8.460*E* + 02**
	PSO-*ω*_3_	1.000*E* + 29(+)	3.161*E* + 30	3.9	1.977*E* + 03
	PSO-*ω*_4_	1.080*E* + 25(+)	3.162*E* + 26	72.1	1.317*E* + 03
	PSO-*ω*_5_	1.000*E* + 19(−)	3.161*E* + 20	0.4	1.827*E* + 03
	PSO-*ω*_6_	2.000*E* + 23(−)	4.468*E* + 24	**84.8**	1.159*E* + 03
	PSO-*ω*_7_	1.030*E* + 25(=)	3.161*E* + 26	0	—
	PSO-*ω*_*cos*_	1.031*E* + 25	3.161*E* + 26	76.4	1.015*E* + 03

The best results are shown in bold.

**Table 8:** Results of the experiment on benchmark functions *f*_1_ ~ *f*_6_.

Benchmark function	Variant	Mean	SD	SR (%)	Average number
*f*_1_	PSO-rand	7.069*E* − 44(=)	7.309*E* − 43	**100**	9.682*E* + 02
	PSO-chaotic	6.130*E* − 44(=)	7.711*E* − 43	**100**	9.693*E* + 02
	PSO-opposition	2.596*E* − 43(+)	6.463*E* − 42	**100**	9.687*E* + 02
	PSO-uniform	**5.674*E*** − **44**	**6.344*E* − 43**	**100**	**9.679*E* + 02**

*f*_2_	PSO-rand	1.167*E* − 44(=)	1.436*E* − 43	**100**	**9.288*E* + 02**
	PSO-chaotic	1.573*E* − 44(+)	2.384*E* − 43	**100**	9.304*E* + 02
	PSO-opposition	**1.056*E*** − **44**(=)	1.637*E* − 43	**100**	9.309*E* + 02
	PSO-uniform	1.157*E* − 44	**1.163*E* − 43**	**100**	9.295*E* + 02

*f*_3_	PSO-rand	3.290*E* − 02(=)	2.851*E* − 02	12.2	**1.359*E* + 03**
	PSO-chaotic	3.547*E* − 02(+)	3.015*E* − 02	11.9	1.387*E* + 03
	PSO-opposition	3.231*E* − 02(=)	**2.805*E* − 02**	11.9	1.378*E* + 03
	PSO-uniform	**3.217*E*** − **02**	2.925*E* − 02	**13.6**	1.361*E* + 03

*f*_4_	PSO-rand	3.935*E* − 17(=)	**1.690*E* − 16**	**100**	**1.356*E* + 03**
	PSO-chaotic	3.705*E* − 17(=)	1.998*E* − 16	**100**	1.358*E* + 03
	PSO-opposition	4.928*E* − 17(+)	3.238*E* − 16	**100**	1.357*E* + 03
	PSO-uniform	**3.601*E*** − **17**	1.704*E* − 16	**100**	1.356*E* + 03

*f*_5_	PSO-rand	1.011*E* + 02(=)	1.484*E* + 03	0	—
	PSO-chaotic	1.105*E* + 02(+)	1.296*E* + 03	**0.2**	**1.908*E* + 03**
	PSO-opposition	1.315*E* + 02(=)	1.763*E* + 03	0	—
	PSO-uniform	**9.081*E*** + **01**	**1.220*E* + 03**	0	—

*f*_6_	PSO-rand	1.020*E* + 27(+)	3.161*E* + 28	64.5	1.814*E* + 03
	PSO-chaotic	1.040*E* + 25(+)	3.161*E* + 26	68.2	1.808*E* + 03
	PSO-opposition	1.010*E* + 21(+)	3.161*E* + 22	**83.8**	**1.794*E* + 03**
	PSO-uniform	**5.061*E*** + **19**	**7.053*E* + 20**	71.4	1.805*E* + 03

The best results are shown in bold.

**Table 9:** Results of the experiment on 20-Dimensional CEC2020 benchmark functions.

No.	Term	PSO	MCJPSO	PSO-DLS	EPSO	HCLPSO	GLPSO	UCPSO
*F*_1_	Mean	7.657*E* + 09(+)	8.746*E* + 04(−)	4.963*E* + 08(+)	2.724*E* + 03(−)	**1.876*E*** + **03**(−)	5.278*E* + 03(−)	1.623*E* + 08
	SD	5.706*E* + 09	1.160*E* + 05	1.125*E* + 09	**1.727*E*** + **03**	1.945*E* + 03	4.114*E* + 03	3.203*E* + 08

*F*_2_	Mean	3.133*E* + 03(+)	3.547*E* + 03(+)	4.748*E* + 03(+)	1.839*E* + 03(−)	**1.480*E*** + **03**(−)	1.631*E* + 03(−)	2.595*E* + 03
	SD	4.421*E* + 02	5.693*E* + 02	3.487*E* + 02	3.804*E* + 02	**1.779*E*** + **02**	2.585*E* + 02	2.013*E* + 02

*F*_3_	Mean	7.972*E* + 02(+)	7.640*E* + 02(+)	7.646*E* + 02(+)	7.502*E* + 02(=)	**7.338*E*** + **02**(−)	7.373*E* + 02(−)	7.505*E* + 02
	SD	6.769*E* + 01	1.579*E* + 01	3.554*E* + 01	1.768*E* + 01	**3.273*E*** + **00**	6.877*E* + 00	8.305*E* + 00

*F*_4_	Mean	2.972*E* + 04(+)	1.905*E* + 03(=)	4.636*E* + 03(+)	1.903*E* + 03(=)	**1.902*E*** + **03**(=)	1.903*E* + 03(=)	1.904*E* + 03
	SD	7.159*E* + 04	1.195*E* + 00	1.150*E* + 04	1.064*E* + 00	**6.273*E*** − **01**	1.613*E* + 00	1.336*E* + 00

*F*_5_	Mean	1.766*E* + 06(+)	3.715*E* + 05(+)	3.620*E* + 05(+)	2.233*E* + 05(+)	2.040*E* + 05(+)	1.875*E* + 05(+)	**1.153*E*** + **05**
	SD	2.832*E* + 06	2.189*E* + 05	3.937*E* + 05	1.786*E* + 05	1.480*E* + 05	2.730*E* + 05	**7.666*E*** + **04**

*F*_6_	Mean	2.212*E* + 03(+)	2.293*E* + 03(+)	1.852*E* + 03(=)	1.617*E* + 03(−)	1.615*E* + 03(−)	**1.603*E*** + **03(−)**	1.898*E* + 03
	SD	2.999*E* + 02	1.341*E* + 02	2.004*E* + 02	1.884*E* + 01	3.539*E* + 01	1.471*E* + 00	**1.314*E*** + **00**

*F*_7_	Mean	6.251*E* + 05(+)	8.681*E* + 04(−)	9.943*E* + 04(=)	8.347*E* + 04(=)	**7.621*E*** + **04**(−)	1.724*E* + 05(+)	8.730*E* + 04
	SD	7.390*E* + 05	6.811*E* + 04	1.245*E* + 05	7.031*E* + 04	**6.545*E*** + **04**	3.510*E* + 05	1.186*E* + 05

*F*_8_	Mean	4.349*E* + 03(+)	2.309*E* + 03(−)	3.839*E* + 03(+)	**2.301*E*** + **03**(−)	**2.301*E*** + **03**(−)	3.234*E* + 03(+)	2.740*E* + 03
	SD	9.377*E* + 02	1.335*E* + 01	1.881*E* + 03	**7.741*E*** **−** **01**	9.830*E* **−** 01	1.241*E* + 03	8.641*E* + 02

*F*_9_	Mean	3.019*E* + 03(+)	2.858*E* + 03(=)	2.896*E* + 03(+)	2.768*E* + 03(−)	**2.745*E*** + **03**(−)	2.868*E* + 03(=)	2.856*E* + 03
	SD	9.117*E* + 01	1.012*E* + 02	2.139*E* + 01	1.166*E* + 02	1.198*E* + 02	1.922*E* + 01	**1.865*E*** + **01**

*F*_10_	Mean	3.181*E* + 03(+)	2.962*E* + 03(=)	2.936*E* + 03(=)	2.948*E* + 03(=)	**2.923*E*** + **03**(−)	2.947*E* + 03(=)	2.947*E* + 03
	SD	2.558*E* + 02	2.810*E* + 01	2.327*E* + 01	2.485*E* + 01	**1.467*E*** + **01**	3.382*E* + 01	3.501*E* + 01

The best results are shown in bold.

**Table 10:** Algorithm complexity of five algorithms on *F*_1_ of 20-dimensional CEC2020 benchmark functions.

Algorithm	*T*_0_ (s)	*T*_1_ (s)	*T*_2_ (s)	(*T*_2_ − *T*_1_)/*T*_0_
PSO	0.014	0.023	0.196	12.357
PSO-DLS	0.014	0.023	0.546	37.357
EPSO	0.014	0.023	0.493	33.571
HCLPSO	0.014	0.023	0.363	24.286
UCPSO	0.014	0.023	0.298	19.643

**Table 11:** CEC2011 real-world optimization problems.

No.	Problems	*D*
*P*_1_	Parameter estimation for frequency-modulated (FM) sound waves	6
*P*_4_	Optimal control of a nonlinear stirred tank reactor	1

**Table 12:** Results on *P*_1_ of CEC2011 real-world optimization problems.

Algorithm	Term	*a*_1_	*ω*_1_	*a*_2_	*ω*_2_	*a*_3_	*ω*_3_	*f*(*x*)
PSO	Best	−1.000	−5.000	−1.500	−4.800	−2.000	4.900	0
UCPSO		1.000	5.000	1.500	−4.800	−2.000	4.900	4.563*E* − 28
PSO	Worst	0.424	4.948	0.698	−2.680	−6.400	6.248	2.333*E* + 01
UCPSO		0.407	−0.992	−2.218	1.160	3.079	−2.667	2.277*E* + 01

**Table 13:** Results on *P*_4_ of CEC2011 real-world optimization problems.

Algorithm	Term	*u*	*f*(*x*)
PSO	Best	1.213	1.433*E* + 01
UCPSO		0.960	1.377*E* + 01
PSO	Worst	1.792	2.108*E* + 01
UCPSO		1.795	2.096*E* + 01

## Data Availability

The data used to support the findings of this study are available from the corresponding author upon request.
